# Patent Foramen Ovale and Ascending Aortic Dilatation Causing Platypnea-Orthodeoxia Syndrome

**DOI:** 10.7759/cureus.26953

**Published:** 2022-07-17

**Authors:** Alaaeldin Ahmed, Arashdeep Rupal, Alexander Walker, Omar Al Omari, Chinmay Jani, Harpreet Singh, Rahul S Nanchal

**Affiliations:** 1 Department of Medicine, Mount Auburn Hospital, Harvard Medical School, Cambridge, USA; 2 Division of Pulmonary and Critical Care, Medical College of Wisconsin, Milwaukee, USA

**Keywords:** right-to-left shunting, hypoxemia, patent foramen ovale, orthodeoxia, platypnea

## Abstract

Platypnea-orthodeoxia syndrome (POS) is an underdiagnosed clinical syndrome characterized by dyspnea (platypnea) and hypoxemia (orthodeoxia) in the upright position that resolves when recumbent. POS is often due to an underlying right-to-left shunt. Four broad mechanisms for the shunt have been described: intracardiac shunts, intrapulmonary shunts, hepatopulmonary syndrome, and pulmonary ventilation-perfusion mismatch.

A 68-year-old male with a past medical history of chronic obstructive pulmonary disease (COPD), obstructive sleep apnea, ascending aortic dilation (3.9 cm), myelofibrosis, and status post stem cell transplant complicated by graft versus host disease was found hypoxemic (oxygen saturation: 82%) on routine visit prompting hospitalization. Hypoxemia initially responded to 40% FiO2 but subsequently progressed to refractory hypoxemia on 100% FiO2. A chest computed tomography (CT) scan showed evidence of multiple segmental pulmonary emboli with patent central pulmonary arteries. Hypoxemia out of proportion to pulmonary embolism clot burden and examination findings consistent with orthodeoxia prompted further investigations. Nuclear medicine scan showed radiotracer activity in both brain and kidneys consistent with a small right-to-left shunt (5.9%). Transesophageal echocardiography (TEE) revealed a patent foramen ovale (PFO) with a right-to-left shunt across the atrial septum, with a maximum opening of 3.5 mm and tunnel length of 25 mm. Right heart catheterization (RHC) is consistent with the right-to-left shunt and normal right heart pressures. The degree of the shunt was not significant enough to explain the degree of hypoxemia, but all the diagnostic studies were performed in a supine position, possibly underestimating the degree of the shunt. PFO closure with transcatheter 30-mm Gore device (GORE® CARDIOFORM, Arizona, USA) decreased supplemental oxygen requirement from 75% high-flow nasal cannula (NC) to room air (RA) immediately after the procedure. The patient was subsequently discharged home on a baseline oxygen requirement of 2 L NC at nighttime.

POS should be suspected when a patient develops severe hypoxemia after changing from a recumbent position to a sitting or standing position. The identification and correction of the shunting or mismatch often allow complete resolution of POS. Transthoracic echocardiography with agitated saline, TEE, and RHC are the diagnosis modalities of choice. Left heart cardiac catheterization remains the gold standard, which would demonstrate a mismatch in oxygen saturation between the pulmonary vein and the aorta. Our patient’s PFO was successfully closed by a percutaneous transcatheter closure device leading to the complete resolution of hypoxemia immediately.

## Introduction

Platypnea-orthodeoxia syndrome (POS) is a rare clinical syndrome characterized by upright dyspnea (platypnea) and hypoxemia (orthodeoxia) that improves when recumbent. The general mechanism underpinning POS involves a fixed or structural abnormality (cardiac or pulmonary) in addition to a functional or dynamic abnormality (increased pulmonary artery pressures, decreased right atrial compliance, and increased venous return), which, under certain circumstances, such as sitting upright, combine to cause oxygen desaturation [1].

The most common structural abnormality referenced is an interatrial shunt, commonly a patent foramen ovale (PFO), atrial septal defect (ASD), or fenestrated atrial aneurysm [1]. Independent of dynamic changes, structural abnormalities alone usually will not cause hypoxemia. Typically, the left atrial pressure exceeds the right atrial pressure and prevents the passage of deoxygenated blood into the systemic circulation. This also explains why, despite a prevalence of approximately 25%-30%, most adults with an isolated PFO are asymptomatic [2]. However, elevated right atrial pressures or mechanical distortion of the cardiac parenchyma, provoked by specific positional changes, may manipulate the defect, causing a right-to-left shunt. Here, we report a case of PFO attributed to dilated ascending aorta presenting as POS.

The abstract of this study was presented at the 2021 American Association of Physicians of Indian Origin (AAPI) Medical Students, Residents, and Fellows section (MSRF) Winter Medical Conference, and the poster was published in Cureus on February 19, 2021.

## Case presentation

**Figure 1:**
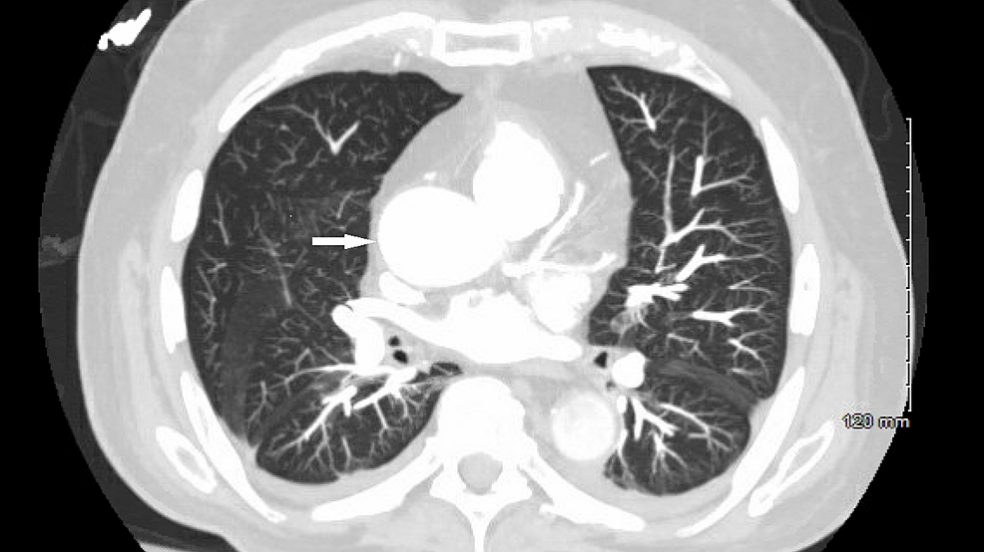
Chest CT (axial view) showing ascending aortic dilation (white arrow).

A 68-year-old male with a past medical history of chronic obstructive pulmonary disease (COPD), obstructive sleep apnea, myelofibrosis, and stem cell transplant complicated by graft versus host disease was found hypoxemic (oxygen saturation of 82% on room air (RA)) on a routine visit prompting hospitalization. Hypoxemia initially responded to 40% oxygen supplementation but subsequently progressed to refractory hypoxemia (oxygen saturation of 83%-85%) on 100% oxygen supplementation. A contrast-enhanced chest computed tomography (CT) demonstrated multiple segmental pulmonary emboli. Central pulmonary arteries remained patent, and the ascending aorta was dilated at 3.9 cm (Figure 1).

However, hypoxemia out of proportion to the degree of pulmonary embolism and examination findings consistent with orthodeoxia (reproducible drop in oxygen saturation from 92%-93% to 82%-83% after changing from recumbent to an upright position) prompted a further workup. The nuclear medicine ventilation-perfusion (V/Q) scan was consistent with a mild right-to-left shunt (5.9%). Transesophageal echocardiography (TEE) revealed a mildly enlarged right atrium and a PFO, with a maximum opening of 3.5 mm and a tunnel length of 25 mm (Videos 1, 2). On right heart catheterization, right-to-left shunt (QP/QS) was calculated at 0.74 with normal right heart pressures. The patient underwent PFO closure with a transcatheter 30-mm Gore device (GORE® CARDIOFORM, Arizona, USA), resulting in the immediate resolution of hypoxemia.

**Video 1:** TEE upper esophageal bicaval view showing a mildly enlarged right atrium and a PFO.

**Video 2:** TEE upper esophageal 60d without Doppler showing a PFO with an opening of 3.5 mm.

## Discussion

Although likely underdiagnosed, the recognition and, therefore, the incidence of POS is increasing [3]. Barriers to diagnosis include the complex nature of the disease and the presence of specific pulmonary/cardiac comorbidities. POS is characterized by upright dyspnea (platypnea) and concomitant arterial desaturation (orthodeoxia), improving in the supine position. A fall in SaO2 of >5% or PaO2 of >4 mmHg upon transitioning from supine to upright/sitting position is essential for diagnosis [4].

In POS, the pathophysiology of arterial desaturation involves mixing deoxygenated venous blood with oxygenated arterial blood through a shunt. Both anatomical and functional aberrations are usually needed for this shunting to occur, which is exaggerated in the upright posture, resulting in the characteristic feature of POS [4]. Anatomical aberrations include intracardiac shunts, intrapulmonary shunts, hepatopulmonary syndrome, ventilation-perfusion mismatch, or a combination of these. In intracardiac shunts, the stretching of atria with upright positioning causes the defect, whether an ASD or PFO, to widen and change orientation with a consequent increase in the shunting of deoxygenated blood. With intrapulmonary shunts, the shunting mechanism increases pulmonary vascular resistance, subsequently increasing right atrial pressure, leading to a reversal of the left-to-right pressure gradient [4,5]. Additionally, pulmonary arteriovenous malformations, often seen in the setting of cirrhosis due to an imbalance in the ratio of pulmonary vasoconstrictors and vasodilators, may result in shunt physiology and the failure to oxygenate venous blood adequately [6]. An upright position may result in further hypoxemia and manifest as POS because the location of the defects is predominantly in the lung bases, and the upright position causes a gravitationally mediated increase in pulmonary blood flow through these defects [7].

Functional aberrations act through a transient reversal of the left-to-right pressure gradient. They can elevate right atrial pressure to the point that the gradient reverses for certain parts of the cardiac cycle, particularly when the patient is standing. This phenomenon may be physiological (posture, inspiration, cough, or Valsalva maneuver) or pathological, producing increased pulmonary vascular resistance or decreased right-sided compliance. Aortic dilatation is a unique example of these functional aberrations, which, in combination with an upright position, may influence an interatrial defect, augmenting venous blood flow to the systemic circulation. The exact mechanism is unknown; however, it is believed that when upright, a dilated aorta may exert mechanical pressure on the interatrial septum, distorting it so that it may increase PFO patency [8-10].

Medina et al. previously described a case in which a 67-year-old female presented with syncope and was found to have a dilated aortic root and variable patency of a PFO seen on tilt-table TEE [8]. In addition, a greater than 15% desaturation was documented from recumbent to supine position. In another case, Shiraishi et al. demonstrated a dramatic increase in trans-atrial mean pressure gradient from 1 mmHg in the supine position to 7 mmHg after sitting. Additionally, a chest CT revealed the presence of a dilated proximal aorta oriented in a horizontal direction, compressing the interatrial septum into the right atrium to lead venous return from the inferior vena cava to the PFO [10]. In both cases, symptoms resolved following the closure of the PFO.

POS should be suspected when a patient develops severe dyspnea or hypoxemia after shifting from a recumbent to a sitting or standing position. Failure of supplemental oxygen to appropriately correct arterial desaturation should also raise suspicion [5]. Tilt-table echocardiography demonstrating variable shunt patency and left and right heart catheterizations with the demonstration of position-dependent variation in transarterial mean pressure gradients help solidify the diagnosis [1]. Once POS is diagnosed, detecting the underlying anatomical defect is crucial. An ultrasound modality mainly diagnoses PFO. Various techniques include transthoracic echocardiography (TTE), TEE, or intracardiac echocardiography (ICE) [2]. Combining these techniques with agitated saline injection and color Doppler imaging (TTE, TEE, and ICE) can detect right-to-left shunt associated with a PFO. Agitated saline bubbles can also help differentiate between intracardiac and intrapulmonary shunts, where detection of the bubbles within three cardiac cycles is noticed in the former. In comparison, five cardiac cycles are noticed in the latter [11]. A diagnosis of the right-to-left shunt can be made when one or more microbubble is seen in the left chambers. A grade 0 shunt is visualizing no microbubble, grade I is visualizing 1-5 microbubbles, grade II is visualizing 6-20 microbubbles, grade III is visualizing 21-50 microbubbles, and grade IV is visualizing >50 microbubbles [12]. Other modalities for assessing intracardiac defects include cardiac catheterization to calculate shunt fraction and demonstrate mismatch in oxygen saturation between the pulmonary vein and the aorta, ventilation‐perfusion scan to demonstrate early extrapulmonary uptake, and cardiac magnetic resonance imaging. If no intracardiac lesion is identified, pulmonary, abdominal, or other conditions known to cause POS should be investigated [5].

Generally, the indications for PFO closure include cryptogenic stroke, systemic embolization, decompression illness, migraine with aura, and finally POS. The definitive treatment for POS secondary to intracardiac shunting involves closure of the interatrial defect facilitating the shunt. Percutaneous closure is now preferred over cardiac surgery to treat ASDs and PFOs, given its decreased morbidity, mortality, and expense. This treatment modality results in symptomatic improvement in >95% of patients. There is also an average increase in upright arterial oxygen saturation of 10%-20% [5]. In our patient, surgical correction of the PFO resulted in near-immediate improvement of his oxygen saturations and respiratory distress. In the absence of definitive surgical treatment, opiate therapy has been shown to have some symptomatic improvement. This is generally reserved for high-risk patients in whom operative intervention may not be tolerated or does not fall within their goals of care [13]. If intrapulmonary shunting is identified, treating the underlying pulmonary disease is the mainstay of management to decrease the V/Q mismatch.

## Conclusions

POS requires an anatomical factor such as interatrial communication secondary to PFO, atrial septal defect or fenestrated atrial septal aneurysm, and a functional factor, which promotes abnormal shunting when the patient rises from a recumbent to an upright position. In our patient, hypoxia in the supine position was thought to result from the anatomical distortion of the right atrium and atrial septum secondary to aortic dilatation, resulting in an increased right-to-left shunt. We also highlight the need to conduct investigations in the upright position before discounting intracardiac shunting as a cause of hypoxia. Cardiac catheterization remains the gold standard to identify mismatch in oxygen saturation between the pulmonary vein and aorta. The identification and correction of the shunting often allow complete resolution of POS.
